# Performance Evaluation of the GeneNAT HHH Real-Time Polymerase Chain Reaction System for Simultaneous Detection of Hepatitis B Virus, Hepatitis C Virus, and Human Immunodeficiency Virus

**DOI:** 10.7759/cureus.106316

**Published:** 2026-04-02

**Authors:** Bhawna Narula, Malika Grover, Reshu Agarwal, Ekta Gupta

**Affiliations:** 1 Department of Clinical Virology, Institute of Liver and Biliary Sciences, New Delhi, IND

**Keywords:** hepatitis b, hepatitis c, hiv, real-time pcr, viral hepatitis

## Abstract

Background

Hepatitis B virus (HBV), hepatitis C virus (HCV), and human immunodeficiency virus (HIV) are major blood-borne pathogens responsible for significant global morbidity and mortality. Accurate and early diagnosis is essential for initiating timely treatment and reducing disease transmission. The World Health Organization has highlighted the need for diagnostic tools that are affordable, sensitive, specific, user-friendly, rapid, and robust, particularly in resource-limited settings. This study evaluates the clinical performance of a combined polymerase chain reaction (PCR)-based detection and viral load quantification assay for HBV DNA, HCV RNA, and HIV RNA (HHH assay) using the GeneNAT 300/340 Real-Time PCR System (Genetix Biotech Asia Pvt. Ltd., India) as the test assay. Its performance was compared with established reference assays: the COBAS® AmpliPrep/COBAS® TaqMan® v2.0 (Roche Diagnostics, USA) for HBV and HCV quantification as comparator assay 1 and the Xpert® HIV Viral Load assay (Cepheid, USA) for HIV RNA quantification as comparator assay 2.

Methodology

A retrospective analysis was conducted using 110 anonymized, de-identified archived plasma samples collected at a tertiary liver care center in India. The cohort comprised a study group of 53 confirmed viral load-positive samples (20 HBV, 18 HCV, and 15 HIV) and a control group of 57 samples negative for HBV, HCV, and HIV infection. For evaluation of diagnostic performance, all archived specimens were tested in parallel using the GeneNAT 300/340 Real-Time PCR System as test assay and the established reference assays: COBAS® AmpliPrep/COBAS® TaqMan® v2.0 for HBV and HCV viral load quantification as comparator assay 1, and the Xpert® HIV Viral Load assay for HIV RNA quantification as comparator assay 2.

Results

The GeneNAT 300/340 Real-Time PCR assay demonstrated 100% concordance with the comparator assays 1 and 2, with no false-positive or false-negative results observed. The assay achieved 100% sensitivity and specificity for all three infections and delivered results within approximately 60 minutes.

Conclusions

The GeneNAT 300/340 Real-Time PCR assay shows significant potential to strengthen diagnostic capacity in low- and middle-income countries. Its ease of use, rapid turnaround time, and robust performance make it a strong candidate for broader implementation, including screening in blood banks and peripheral healthcare settings as part of a decentralized diagnostic approach. However, larger-scale prospective studies are required to further establish its clinical utility, cost-effectiveness, and feasibility for widespread adoption.

## Introduction

Hepatitis B virus (HBV), hepatitis C virus (HCV), and human immunodeficiency virus (HIV) pose major public health challenges and contribute substantially to the global disease burden, particularly in resource-limited settings. According to the World Health Organization (WHO) Global Hepatitis Report 2024, India ranks second worldwide in viral hepatitis burden, with an estimated 29.8 million individuals infected with HBV and 5.5 million infected with HCV [1]. Data from the National AIDS Control Organisation (NACO) estimate that approximately 2.5 million people were living with HIV (PLHIV) in India as of 2023 [2].

The WHO Global Health Sector Strategies (WHO-GHSS) 2022-2030 aim to eliminate viral hepatitis and acquired immunodeficiency syndrome (AIDS) as public health threats by 2030 [3]. India’s National AIDS and STD Control Programme Phase V (2021-2026) emphasizes early diagnosis, expansion of testing coverage, and decentralized access to quality-assured molecular diagnostics [4]. The global hepatitis elimination strategy targets a 90% reduction in new infections and a 65% reduction in mortality by 2030. In parallel, the UNAIDS 95-95-95 targets aim for 95% of PLHIV to know their status, 95% of those diagnosed to receive antiretroviral therapy, and 95% of those on treatment to achieve viral suppression [5].

A key factor in eliminating these viral infections is effective screening and testing using sensitive and specific point-of-care (POC) methods, followed by confirmation through molecular viral load testing, particularly in peripheral areas where healthcare infrastructure is limited [6]. The WHO has recently recommended innovative diagnostic strategies such as POC molecular testing and dried blood spot testing to expand testing coverage and reduce the diagnostic window period [7].

Blood transfusion remains a significant route of transmission of these viruses in developing and resource-constrained settings where nucleic acid amplification testing (NAT) is not routinely implemented. In India, the Drugs and Cosmetics Act (1940) mandates screening of donated blood for HBV, HCV, and HIV; however, disparities in testing methodologies and NAT adoption persist across blood centers [8]. Unlike developed countries, where NAT is standard practice, reliance on serological testing alone in many Indian settings risks missing early or occult infections during the window period [9].

Currently available molecular platforms are often limited by high costs, operational complexity, batch-based workflows leading to prolonged turnaround times (TATs), and the need for highly trained personnel, factors that restrict their widespread use in resource-limited environments. Therefore, affordable, rapid, and user-friendly molecular diagnostic solutions are urgently needed. The newly introduced POC system, Smart Sure™ HHH Real-Time PCR Kit, operating on the GeneNAT 300/340 platform, enables simultaneous qualitative detection of HBV DNA, HCV RNA, and HIV RNA, significantly reducing diagnostic turnaround time while maintaining high analytical performance.

In the present study, the primary objective was to evaluate the clinical performance of the GeneNAT 300/340 Real-Time PCR assay (Genetix Biotech Asia Pvt. Ltd., India) for the simultaneous detection of the HBV DNA, HCV RNA, and HIV RNA, hereafter referred to as the test assay. The secondary objective was to determine the sensitivity, specificity, and concordance of the GeneNAT 300/340 Real-Time PCR assay against established reference methods: COBAS® AmpliPrep/COBAS® TaqMan® v2.0 (Roche Diagnostics, USA) for HBV DNA and HCV RNA as comparator assay 1 and Xpert® HIV Viral Load assay (Cepheid, USA) for HIV RNA as comparator assay 2.

## Materials and methods

Study population and specimens

**Figure 1:**
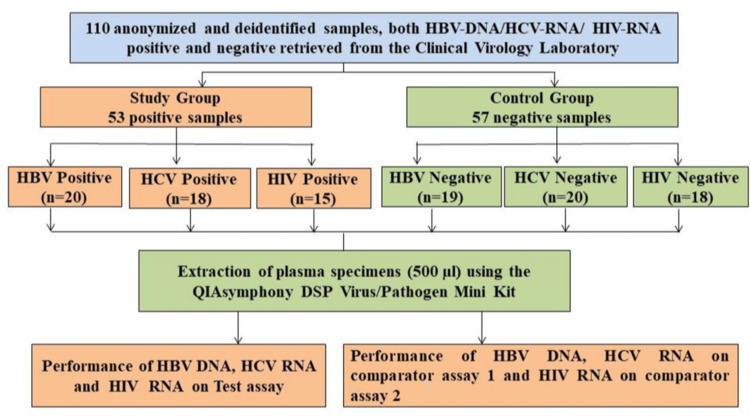
Flowchart depicting the study workflow, including sample selection, categorization into study and control groups, and testing of samples. HBV = hepatitis B virus; HCV = hepatitis B virus; HIV = human immunodeficiency

This retrospective study was conducted in the Department of Clinical Virology at a tertiary care liver center. A total of 110 de-identified, anonymized, archived clinical plasma samples that had undergone a single freeze-thaw cycle were included. All samples were residual specimens from routine diagnostic testing of patients attending the outpatient department or admitted to the hospital, retrieved from the laboratory repository. The 110 plasma specimens were stratified into two groups, namely, study and control cohorts, as illustrated in Figure 1.

In this study, 53 (48%) specimens were categorized into the study group and 57 (52%) into the control group. The study group comprised samples with confirmed viral load positivity based on reference assay and was further subdivided as follows: HBV-positive samples (n = 20, 37.7%) were defined as specimens positive for HBsAg, total anti-HBc, and HBV DNA [10]; HCV-positive (n = 18, 33.9%) samples were defined as specimens positive for both anti-HCV antibodies and HCV RNA [11]; and HIV-positive samples (n = 15, 28.3%) were defined as specimens positive for both anti-HIV-1/2 antibodies and HIV RNA [12]. The control group (n = 57) consisted of specimens that tested negative for both serological markers (HBsAg, total anti-HBc, anti-HCV, and anti-HIV-1/2) and molecular markers (HBV DNA, HCV RNA, and HIV RNA) for HBV, HCV, and HIV, respectively. These samples were obtained from apparently healthy blood donors.

Demographic and relevant clinical details were obtained from the Hospital Information System. Samples with insufficient volume and those from patients with HBV, HCV, or HIV co-infections were excluded from the study. All included plasma specimens were retrieved from −80°C storage, and it was ensured that each sample had undergone only a single freeze-thaw cycle. All retrieved samples were tested in parallel on both the test assay and the comparator assay in the same freeze-thaw cycle.

Ethical approval for the study was obtained from the Institutional Ethics Committee (IEC). Given the retrospective design and the use of de-identified, archived residual plasma samples, the requirement for individual informed consent was waived.

Performance of the test assay (Smart Sure™ HHH Qualitative Real-Time PCR)

Viral Nucleic Acid Extraction

Viral nucleic acids were extracted from 500 µL of plasma specimens with the final elution volume of 90 µL using the QIAsymphony DSP Virus/Pathogen Mini Kit on the QIAsymphony SP instrument (Qiagen, Germany) according to the manufacturer’s instructions.

GeneNAT-300/340 Real-Time PCR System

**Figure 2:**
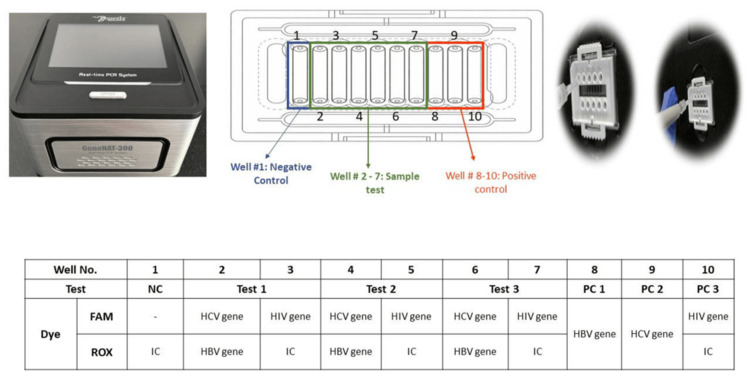
GeneNAT 300/340 Real-Time PCR instrument and Smart Sure™ HHH qualitative Real-Time PCR assay chip used for the simultaneous detection of HBV, HCV, and HIV. HBV = hepatitis B virus; HCV = hepatitis B virus; HIV = human immunodeficiency; PCR = polymerase chain reaction; NC = negative control; PC = positive control; IC = internal control

The plasma samples in the study were analyzed on the test assay for the qualitative detection of HBV DNA, HCV RNA, and HIV RNA as per the manufacturer’s recommended operating conditions. The system utilizes a biochip-based format containing 10 wells, enabling the simultaneous testing of three samples for the nucleic acids of the three viruses, while the remaining wells are designated for negative control, positive controls, and internal control, as depicted in Figure 2. The test was performed by adding 10 µL of master mix and 10 µL of extracted viral nucleic acid to the designated wells on the chip, followed by sealing with the adhesive tapes provided in the test kit. Reverse transcription on the chip was under the following temperature conditions: 50°C for 600 seconds (one cycle), followed by an initial denaturation at 95°C for 180 seconds, and subsequent cycling with denaturation at 95°C for 8 seconds and annealing/extension at 60°C for 35 seconds, with fluorescence data acquisition at the end of each annealing/extension step. Upon completion of the run, results were automatically displayed by the system within approximately 60 minutes. The assay has a limit of detection (LOD) of 12.00 IU/mL for HBV, 31.25 IU/mL for HCV, and 31.51 IU/mL for HIV, calibrated against the 4th WHO HBV standard (NIBSC: 10/266), the 6th WHO HCV standard (NIBSC: 18/184), and the WHO HIV standard (NIBSC: 16/194), respectively.

Performance using the comparator assays 1 and 2

HBV DNA and HCV RNA were simultaneously detected using the COBAS® AmpliPrep/COBAS® TaqMan® HBV/HCV Test v2.0 (Roche Diagnostics, USA), a fully automated system for nucleic acid extraction and real-time polymerase chain reaction (PCR) amplification, in accordance with the manufacturer’s instructions. The LODs for HBV and HCV were 9 IU/mL and 15 IU/mL, respectively. For HIV RNA quantification, samples were tested using the Xpert® HIV Viral Load assay on the GeneXpert® system (Cepheid, USA), which has an LOD of 18.3 copies/mL.

Statistical analysis

The diagnostic accuracy of the GeneNAT 300/340 Real-Time PCR assay for the detection of HBV, HCV, and HIV viremia was evaluated by calculating sensitivity and specificity with 95% confidence intervals (CIs). Continuous variables were expressed as mean ± standard deviation (SD) or median with interquartile range (IQR), as appropriate. Categorical variables were presented as percentages.

## Results

A total of 110 subjects were enrolled in the study, with a slight female predominance observed. The detailed baseline demographic characteristics and viral load distribution of the study population (n = 110), as determined by the comparator assays 1 and 2, are presented in Table 1.

**Table 1:** Baseline demographic characteristics and viral load distribution of the study population (n = 110) as determined by the comparator assays 1 and 2. HBV = hepatitis B virus; HCV = hepatitis B virus; HIV = human immunodeficiency; IQR = interquartile range; SD = standard deviation

VARIABLES	Value
Patients, n	110
Male:female ratio	0.93:1
Age in years, mean ± SD	47.2 ± 14.6
HBV DNA median (IQR) (log₁₀ IU/mL)	4.58 (3.22–5.73)
HCV RNA median (IQR) (log₁₀ IU/mL)	5.20 (4.21–6.01)
HIV-1 RNA median (IQR) (log₁₀ copies/mL)	4.27 (4.21–4.98)
HBV DNA range (log₁₀ IU/mL)	1.70–5.92
HCV RNA range (log₁₀ IU/mL)	1.60–6.78
HIV RNA range (log₁₀ copies/mL)	3.58–5.11

Clinical performance of the Smart Sure™ HHH Qualitative Real-Time PCR

An overall concordance of 100% was observed between the test assay and the comparator assays 1 and 2. The test assay demonstrated a sensitivity of 100% (95% CI = 94.3%-100%), as shown in Table 2. All samples in the control group tested negative with the test assay, resulting in a specificity of 100% (95% CI = 94.7%-100%).

**Table 2:** Performance of the Smart Sure™ HHH Qualitative Real-Time PCR Kit compared with the comparator assays 1 and 2. Confidence intervals were calculated using the exact binomial (Clopper–Pearson) method.

Parameter	Reference: Positive (n)	Reference: Negative (n)	Result	95% confidence interval
Sensitivity	53	-	100%	94.3%-100%
Specificity	-	57	100%	94.7%-100%

## Discussion

The present study evaluated the performance characteristics of the newly introduced Smart Sure™ HHH Real-Time PCR Kit performed on the GeneNAT 300/340 (test assay), a near POC molecular diagnostic platform designed for the simultaneous qualitative detection of HBV, HCV, and HIV (comparator assays 1 and 2). The test assay results were compared with established quantitative comparator assays 1 and 2 using plasma specimens representing a broad range of viral loads. Across this spectrum of clinical samples, the test assay demonstrated 100% concordance with the comparator assays 1 and 2 in detecting the presence or absence of viral nucleic acids, indicating that it is a reliable and promising diagnostic tool. The test was simple to perform and required minimal laboratory infrastructure, primarily limited to nucleic acid extraction, making it a feasible option for decentralized settings with limited access to molecular diagnostics. While previous studies have validated individual near POC platforms such as GeneXpert for HBV and HCV detection [13,14], there is limited literature on the implementation of triplex molecular assays in resource-limited environments. The role of NAT is particularly important in transfusion medicine, as it reduces the diagnostic window period and enhances blood safety [15]. In India, HBV remains the most prevalent transfusion-transmitted infection, with reported prevalence among blood donors ranging from 0.49% to 1.61% [16].

Globally, automated triplex NAT assays, such as the Procleix series (Gen-Probe-Novartis, USA) and COBAS TaqScreen MPX v1.0/v2.0 (Roche Molecular Systems, USA), are recognized for their high sensitivity, specificity, and robustness [17]. However, these platforms are cost-intensive, require centralized laboratory infrastructure, and depend on highly trained technical personnel. Even newer random-access platforms such as NeuMoDx (NeuMoDx Molecular, Inc., a QIAGEN company, Ann Arbor, MI, USA), which have demonstrated excellent concordance with standard methods [18], face similar constraints. Their high installation and maintenance costs continue to limit adoption in peripheral or resource-limited settings, underscoring the need for affordable and user-friendly alternatives such as the HHH Real-Time PCR Kit.

Given this landscape, there is a critical need for affordable, user-friendly, and decentralized molecular diagnostic platforms capable of delivering rapid, sensitive, and specific detection of multiple pathogens in a single run, particularly in low- and middle-income countries [19]. The Smart Sure™ HHH assay, operated on the GeneNAT 300/340 platform, effectively addresses this gap through a microfluidic chip-based approach that combines the analytical sensitivity of conventional systems with simplified test performance.

Cost remains a major barrier to the widespread implementation of POC molecular assays. In this context, the GeneNAT system offers a substantial advantage, with lower capital investment and reduced operational costs compared to traditional platforms. Its ease of use, minimal training requirements, and rapid TAT (less than one hour post-extraction) make it particularly suitable for integration into district hospitals, peripheral laboratories, and blood banks, thereby expanding access to high-quality molecular diagnostics in resource-constrained settings.

Despite these promising findings, this study has certain limitations. It was a single-center evaluation conducted using archived clinical specimens under controlled laboratory conditions, which may not fully reflect the challenges encountered in routine clinical or field settings. Further studies are needed to determine the reproducibility, operational performance, and sustainability of the assay, particularly in resource-constrained environments. To establish broader applicability and cost-effectiveness, future prospective multicenter studies in real-world healthcare settings are warranted.

## Conclusions

The integration of molecular testing at the POC represents a pivotal advancement in the decentralization of diagnostic services, particularly in low-income settings and for blood product screening. The GeneNAT 300/340-based Smart Sure™ HHH RT-PCR Kit (test assay) demonstrated high sensitivity and specificity when compared with established comparator assays 1 and 2, such as the COBAS® AmpliPrep/COBAS® TaqMan® v2.0 and the Cepheid Xpert HIV Viral Load assay. These encouraging initial findings suggest that this single multiplex assay could significantly streamline diagnostic workflows and support timely clinical decision-making. However, larger prospective multicenter studies are required to further establish its clinical utility, cost-effectiveness, and scalability across diverse healthcare settings.
